# Quinolones: from antibiotics to autoinducers

**DOI:** 10.1111/j.1574-6976.2010.00247.x

**Published:** 2010-08-25

**Authors:** Stephan Heeb, Matthew P Fletcher, Siri Ram Chhabra, Stephen P Diggle, Paul Williams, Miguel Cámara

**Affiliations:** School of Molecular Medical Sciences, Centre for Biomolecular Sciences, University Park, University of NottinghamNottingham, UK

**Keywords:** quorum sensing, quinolone, quinoline, *Pseudomonas*, *Burkholderia*, virulence

## Abstract

Since quinine was first isolated, animals, plants and microorganisms producing a wide variety of quinolone compounds have been discovered, several of which possess medicinally interesting properties ranging from antiallergenic and anticancer to antimicrobial activities. Over the years, these have served in the development of many synthetic drugs, including the successful fluoroquinolone antibiotics. *Pseudomonas aeruginosa* and related bacteria produce a number of 2-alkyl-4(1*H*)-quinolones, some of which exhibit antimicrobial activity. However, quinolones such as the *Pseudomonas* quinolone signal and 2-heptyl-4-hydroxyquinoline act as quorum-sensing signal molecules, controlling the expression of many virulence genes as a function of cell population density. Here, we review selectively this extensive family of bicyclic compounds, from natural and synthetic antimicrobials to signalling molecules, with a special emphasis on the biology of *P. aeruginosa*. In particular, we review their nomenclature and biochemistry, their multiple properties as membrane-interacting compounds, inhibitors of the cytochrome *bc*_1_ complex and iron chelators, as well as the regulation of their biosynthesis and their integration into the intricate quorum-sensing regulatory networks governing virulence and secondary metabolite gene expression.

## Introduction

**Fig. 1:**
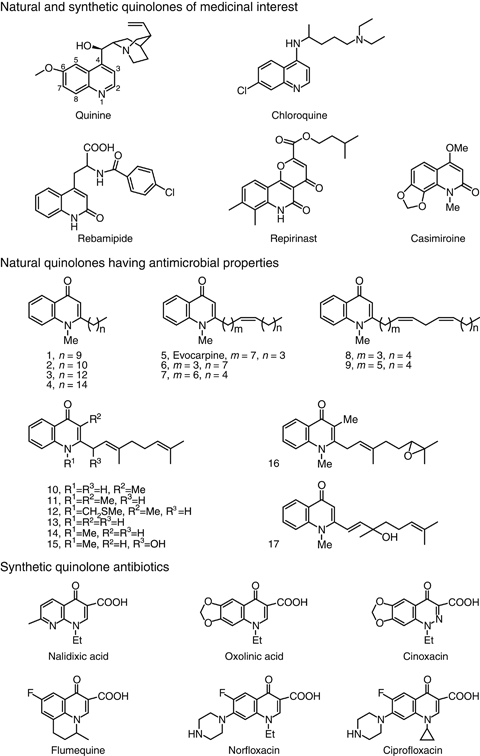
Natural and synthetic quinolones of medicinal interest, quinolone antibiotics. Several plant, animal and microbial species produce quinolone compounds of medicinal interest such as the antimalarial quinine extracted from *Cinchona* spp., or the 2-quinolone casimiroine, an antimutagen extracted from *Casimiroa edulis*. Among the synthetic 2-quinolones are the antiulcer agent rebamipide and the antihistamine, repirinast. Naturally occurring quinolones having antimicrobial activities such as evocarpine and related compounds (nos 1–17) produced by *Evodia rutaecarpa* are active against *Helicobacter pylori*, a causative agent of peptic ulcers and gastric cancer. The quest for synthetic analogues of quinine led to the discovery of nalidixic acid, oxolinic acid and cinoxacin, and then to the development of an extensive family of fluoroquinolone antibiotics such as flumequine, norfloxacin and ciprofloxacin. The heteroaromatic ring atom numbering common to all quinolones is indicated for quinine.

Quinolones are molecules structurally derived from the heterobicyclic aromatic compound quinoline, the name of which originated from the oily substance obtained after the alkaline distillation of quinine (Gerhardt, 1842). Since the isolation of quinine from *Cinchona* bark in 1811, many other quinoline derivatives have been isolated from natural sources (Fig. 1). In particular, 2-hydroxyquinoline and 4-hydroxyquinoline, which predominantly exist as 2(1*H*)-quinolone and 4(1*H*)-quinolone, respectively, and form the core structure of many alkaloids, were isolated from plant sources. Several different animal and bacterial species also produce compounds of the quinolone class. These differ not only in the varied substitutions in the carbocyclic and heteroaromatic rings but also have other rings fused to the quinolone nucleus. These have been reviewed on a yearly basis by J.P. Michael in Natural Product Reports (Michael, 2008). Some of these naturally occurring quinolones have profound medicinal properties while others have served as lead structures and provided inspiration for the design of synthetic quinolones as useful drugs. For example (Fig. 1), among 2-quinolones, rebamipide is an antiulcer agent and repirinast has antihistamine properties useful in the treatment of allergic asthma (Uchida *et al*., 1987). While screening compounds for potential cancer chemopreventive properties, casimiroine, isolated from the seeds of *Casimiroa edulis*, was found to have antimutagenic activity (Ito *et al*., 1998). Several 4-quinolone alkaloids, mainly isolated from plant and microbial sources, have antimicrobial activity. For example, 2-alkyl-4(1*H*)-quinolones (AQs) (Fig. 1, compounds 1–4) and 1-methyl-2-[(4*Z*)-tridecenyl]-4(1*H*)-quinolone, evocarpine, its structural isomers and unsaturated homologues (Fig. 1, compounds 5–9) isolated from the extracts of *Evodia rutaecarpa* show antibacterial activity against *Helicobacter pylori*, which is implicated in the pathogenesis of chronic gastritis, peptic ulcers and gastric cancers (Rho *et al*., 1999; Hamasaki *et al*., 2000;). The alkaloid 1 shown in Fig. 1 is rather rare as it bears *n*-decyl, an even number of carbons in the 2-position. Also, no fewer than eight further 4-quinolones (Fig. 1, compounds 10–17) isolated from the fermentation broth of the actinomycete *Pseudonocardia* spp. CL38489 are active in inhibiting the growth of *H. pylori*. The most potent compound is the epoxide (Fig. 1, compound 16), which has a potent bactericidal [minimal inhibitory concentration (MIC) 10 ng mL^−1^] and an even more pronounced bacteriostatic effect (MIC 0.1 ng mL^−1^) (Dekker *et al*., 1998). These quinolones are characterized by the presence of a geranyl or oxidized geranyl side chain at C-2 in place of the usual fatty acid-derived alkyl or alkenyl chain normally found in microbial quinolones. The screening of synthetic analogues of quinine for novel antiplasmodial drugs led to the serendipitous discovery of a precursor used in the synthesis of chloroquine, 7-chloroquinoline, which exhibited antimicrobial activities *in vitro*. Further investigation of this and similar compounds such as the structurally related 1,8-naphthyridones (which are quinolones with a nitrogen atom substituting C-8) resulted in the discovery of nalidixic acid (1-ethyl-7-methyl-4-oxo-1,8-naphthyridine-3-carboxylic acid), which was to become the first practical synthetic quinolone antibiotic (Lesher *et al*., 1962). This rapidly led to the development of several other 4-quinolone-based antibiotics such as oxolinic acid, cinoxacin and flumequine (Fig. 1), used clinically to treat Gram-negative bacterial infections, and later on to second-generation drugs such as norfloxacin and ciprofloxacin (Fig. 1), also effective against some Gram-positive bacteria. All of the quinolone antibiotics are characterized by the presence of a carboxylic acid function at C-3 (Rohlfing *et al*., 1976; Mårdh *et al*., 1977; Barry *et al*., 1984; Galante *et al*., 1985; Aboul-Fadl & Fouad, 1996;).

**Fig. 2:**
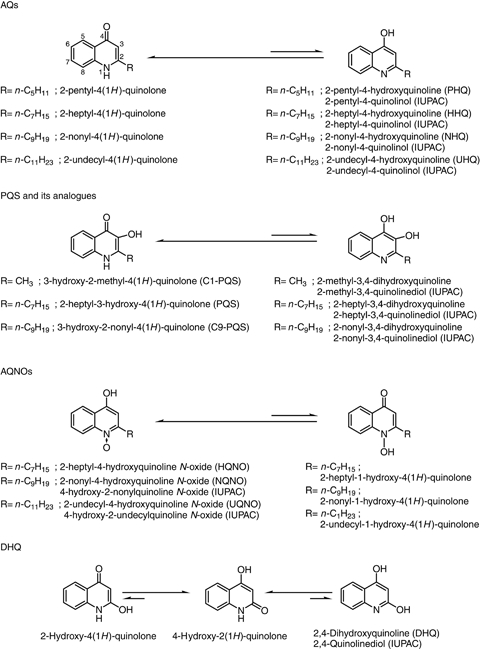
Structure, IUPAC names and abbreviations of AQ molecules synthesized by *Pseudomonas aeruginosa* and a synthetic analogue. Both the tautomeric lactam and the phenolic forms of each molecule are shown. Arrows indicate the equilibrium of these molecules as would exist under physiological conditions. Where more than one name exists for a molecule, the IUPAC designation is indicated, although this may not be the nomenclature used most frequently. The compound C1-PQS is a synthetic analogue that is not produced by *P. aeruginosa*.

Interest in the antipathogenic properties of common bacteria started with the pioneering work of Louis Pasteur. Notably, in 1877, Pasteur reported that the coinoculation of *Bacillus anthracis* with other common living bacteria in animals prevented the development of anthrax, when septicaemia could be avoided. This was interpreted, following the idea that ‘life can prevent life’, as being the result of a competition for oxygen (Pasteur & Joubert, 1877). After Emmerich and Pawlowsky were able to prevent the development of anthrax in preinfected rabbits and guinea-pigs by the inoculation of *Streptococcus* spp., Charles Bouchard reproduced this effect in 1889 with pure cultures of *Bacillus pyocyaneus* (*Pseudomonas aeruginosa*) (Bouchard, 1889). Ten years later, in 1899, Rudolf Emmerich and Oscar Löw concluded that *P. aeruginosa* released an active antibacterial substance into the medium after cell-free preparations from this organism were found to be sufficient to prevent the development of anthrax, and as it was thought to be the result of an enzymatic process, they called it *pyocyanase* (Emmerich & Löw, 1899). In 1945, this preparation was determined to consist of a mixture of heat-stable compounds that were separated, partially characterized and named the Pyo compounds (Hays *et al*., 1945). Pyo I–IV, which later were found to be AQs (Fig. 2), presented strong antibacterial activities against Gram-positive organisms, although much less against Gram-negative bacteria, with Pyo II being 10 times more potent compared with the others. However, Pyo II was toxic and ineffective at protecting mice at subtoxic doses against *Streptococcus pneumoniae* or *Mycobacterium tuberculosis* infections (Wells *et al*., 1947).

## Natural antimicrobial quinolones

In addition to having antimicrobial activity *in vitro*, the Pyo compounds produced by *P. aeruginosa* were found to antagonize, under certain conditions, the action of streptomycin and dihydrostreptomycin against Gram-positive bacteria (Lightbown, 1950, 1954). This inhibitory activity was rapidly attributed to Pyo II, which is a mixture of 2-alkyl-4-hydroxyquinoline *N*-oxides (AQNOs) (Cornforth & James, 1954, 1956; Lightbown & Jackson, 1954). The inhibitory activity of these *N*-oxides (at concentration ratios with respect to streptomycin of the order of 1 : 100) was found to correlate with the potent inhibition of electron transport in both heart-muscle and bacterial cells through the cytochrome *bc*_1_ segment (ubiquinol:cytochrome *c* oxidoreductase) of the respiratory chain (Lightbown & Jackson, 1954, 1956). This is in line with the need for respiration and the transmembrane potential required for bacteria to take up aminoglycoside antibiotics (Hancock, 1962; Damper & Epstein, 1981; Arrow & Taber, 1986; Taber *et al*., 1987;). 2-Heptyl-4-hydroxyquinoline *N*-oxide (HQNO) acts as a ubiquinone and menaquinone analogue on quinone-reactive cytochrome *b* enzymes in various organisms (Van Ark & Berden, 1977; Smirnova *et al*., 1995; Rothery & Weiner, 1996;). The antimicrobial effect of the *N*-oxides appears to be limited to Gram-positive bacteria and offers an explanation as to how *P. aeruginosa* becomes the dominant species over *Staphylococcus aureus* in cystic fibrosis (CF) lung infections (Machan *et al*., 1992), although additional factors such as pyocyanin, cyanide and *N*-(3-oxododecanoyl)-l-homoserine lactone have been shown to play a similar role (Qazi *et al*., 2006; Voggu *et al*., 2006;). Furthermore, long-term exposure of *S. aureus* to physiological concentrations of HQNO selects for aminoglycoside-resistant, small-colony variants that are typically found in chronic lung infections (Hoffman *et al*., 2006; Biswas *et al*., 2009;). Interestingly, the formation of *S. aureus* small-colony variants is the first step towards the development of dual target resistance against fluoroquinolones (Pan *et al*., 2002). The low *in vivo* efficacy, combined with the strong toxicity on mitochondrial respiration, prevented the development of AQNOs as therapeutic antibiotics. However, due to its interference with quinone-dependent cytochromes, HQNO became an invaluable reagent for the study of electron transport chains.

**Fig. 3:**
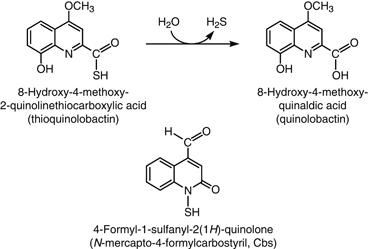
Sulphur-containing quinolones produced by some pseudomonads. Thioquinolobactin, a compound exhibiting strong antifungal properties, is produced by *P. fluorescens* ATCC 17400. Upon spontaneous hydrolysis, thioquinolobactin is rapidly converted into quinolobactin, which then acts as a siderophore. *Pseudomonas fluorescens* G308 produces Cbs, which also exhibits potent fungicidal properties.

A number of quinolones with interesting antimicrobial properties are also produced by various pseudomonads and other microorganisms. For example, under iron limitation, *Pseudomonas fluorescens* ATCC 17400 produces quinolobactin (8-hydroxy-4-methoxyquinaldic acid, Fig. 3), which acts as a siderophore (Mossialos *et al*., 2000). Quinolobactin results from the rapid hydrolysis of the precursor molecule 8-hydroxy-4-methoxy-2-quinolinethiocarboxylic acid (thioquinolobactin), which, as opposed to quinolobactin, has strong antifungal activity against the plant pathogen *Pythium debaryanum* (Matthijs *et al*., 2007). Thioquinolobactin is synthesized via a unique pathway from l-tryptophan via xanthurenic acid and sulphurylation by QbsE, a small sulphur carrier protein (Matthijs *et al*., 2004; Godert *et al*., 2007;). *Pseudomonas fluorescens* G308, a potential biocontrol strain, produces *N*-mercapto-4-formylcarbostyril [Cbs, 4-formyl-1-sulphanyl-2(1*H*)-quinolone, Fig. 3], a quinolone that contains an unusual *N*-mercaptoamide functional group and that has strong antifungal properties against plant pathogens such as *Fusarium* spp., *Cladosporium cucumerinum* and *Colletotrichum lagenarium* (Fakhouri *et al*., 2001). Although the biosynthetic pathway for Cbs formation has not yet been elucidated, it was suggested that this compound may be derived from AQs produced via a similar biochemical pathway as that in *P. aeruginosa*. The marine bacteria *Pseudomonas bromoutilis* (Wratten *et al*., 1977) and *Alteromonas* strain SWAT5 (Long *et al*., 2003) both synthesize 2-pentyl-4-quinolone (PHQ, also called 2-*n*-pentyl-4-quinolinol) and 2-heptyl-4-hydroxyquinoline (HHQ, also called 2-*n*-heptyl-4-quinolinol), which were identified as a consequence of their antibacterial activities. PHQ inhibits the growth of cyanobacteria (*Synechococcus*), algae (*Chaetoceros simplex*, *Cylindotheca fusiformis* and *Thalassiosira weissflogii*) and impacts on particle-associated marine bacterial communities (Long *et al*., 2003). HHQ and PHQ have antibacterial activity against *Vibrio anguillarum*, *S. aureus*, *Candida albicans* and *Vibrio harveyi* (Wratten *et al*., 1977). From sponge-associated marine pseudomonads, several other AQs with various substitutions have been identified and appear to have antibacterial, antiplasmodial, antiviral or cytotoxic properties (Debitus *et al*., 1998; Bultel-Poncé*et al*., 1999;). The obligate aerobic yeast *Yarrowia lipolytica* produces 1-hydroxy-2-dodecyl-4(1*H*)-quinolone, a potent inhibitor of the alternative NADH:ubiquinone oxidoreductases, which acts as a ubiquinone analogue (Eschemann *et al*., 2005), an activity reminiscent of the quinolone *N*-oxides produced by *P. aeruginosa*, which act on the cytochrome *bc*_1_ complex. In addition, a large number of additional quinoline alkaloids produced by a variety of other microorganisms, plants and animals have been discovered every year (annually reviewed by J.P. Michael), of which the majority still have to be studied with respect to their biological properties. However, as all the natural quinolones described so far lack the 3-carboxy group, which is essential for the binding and blocking of DNA-type IIA topoisomerase complexes, the antibacterial mechanism of action of these compounds remains to be elucidated.

## Synthetic quinolone antibiotics

The practical applications of nalidixic acid (Fig. 1) as an antimicrobial of therapeutic interest became evident soon after it was discovered (Lesher *et al*., 1962; Ward-Mcquaid *et al*., 1963;). Because it is a polar molecule that avidly conjugates to serum proteins, and therefore presents a large volume of distribution, it is inadequate for the systemic treatment of infections. However, both nalidixic acid and its principal 7-hydroxymethyl metabolite that remains active undergo rapid renal excretion and readily accumulate in the urinary tract (Rollo, 1966; Van Bambeke *et al*., 2005;). As nalidixic acid is notably efficient at arresting the growth of common enterobacteria, its principal indication was in the treatment of uncomplicated urinary tract infections (Ward-Mcquaid *et al*., 1963). It is, however, of little use against infections occurring outside of the urinary tract or those that are caused by organisms such as *P. aeruginosa* and Gram-positive pathogens that are intrinsically resistant to the practical therapeutic concentrations of the antibiotic. From the 1980s onwards, there appeared successive generations of antibiotics related to nalidixic acid such as the fluoroquinolones, which, due to substitutions in the molecule, more specifically the addition of a 6-fluoro group, have extended therapeutic spectra and enhanced pharmacokinetic properties. The development of fluoroquinolones (Fig. 1) such as flumequine, norfloxacin and ciprofloxacin (one of the most consumed antibiotic worldwide; Ruiz, 2003) extended the spectrum of activity of quinolone antibiotics against infections caused by a variety of otherwise resistant organisms such as *P. aeruginosa* and both aerobic and anaerobic Gram-positive pathogens, and enabled the treatment or the prevention of more severe conditions such as renal, respiratory, abdominal and sexually transmitted bacterial infections (for an extensive review on quinolone antibiotics, see Van Bambeke *et al*., 2005).

Quinolone antibiotics act by inhibiting the two type IIA bacterial topoisomerases: DNA gyrase and topoisomerase IV (bacterial type IIA topoisomerases have been reviewed recently by Sissi & Palumbo, 2010). DNA gyrase is a heterotetramer formed by two subunits encoded by *gyrA* (*nalA*) and *gyrB* (*nalC*). GyrA together with GyrB acts by creating DNA gates or double-stranded gaps in the DNA through which the strands are passed, introducing negative supercoils into DNA and relaxing the positive supercoiling resulting from replication as the strands unwind (Champoux, 2001; Wang, 2002; Corbett & Berger, 2004; Leo *et al*., 2005; Drlica *et al*., 2009;). Nalidixic and oxolinic acid, a more potent, but structurally similar quinolone antibiotic (Staudenbauer, 1976), were initially found to inhibit *in vitro* the supercoiling activity of purified DNA gyrase (Gellert *et al*., 1977; Sugino *et al*., 1977;). Whereas only gyrase is able to introduce negative supercoiling, the function of DNA topoisomerase IV, a heterotetramer formed by two ParC-ParE subunits similar to the GyrA-GyrB subunits of DNA gyrase, is essential for the relaxation of supercoiled DNA and the resolution of catenated DNA molecules after replication (Kato *et al*., 1990, 1992; Adams *et al*., 1992; Peng & Marians, 1993; Hoshino *et al*., 1994; Chen *et al*., 1996). The precise mode of action of the quinolone antibiotics on type IIA topoisomerases has long been debated. However, crystallographic studies strongly suggest that these molecules essentially act by blocking the DNA–topoisomerase complexes when the nucleic acid is cleaved (Chen *et al*., 1996; Laponogov *et al*., 2009;). Thus, in addition to sharing structural and functional similarities, both DNA gyrase and topoisomerase IV can be inhibited by quinolone antibiotics, leading to bacterial cell death due to chromosome fragmentation. Whereas the target of quinolone antibiotics in *Escherichia coli* and other Gram-negative bacteria is mainly DNA gyrase, in Gram-positive species such as *S. aureus* or *S. pneumoniae*, their principal mode of action lies mainly in the inhibition of topoisomerase IV, with exceptions depending on the particular fluoroquinolone compound and bacterial species (Higgins *et al*., 2003; Eliopoulos, 2004; Laponogov *et al*., 2009;).

Resistance to quinolone antibiotics (reviewed by Jacoby, 2005 and by Martinez *et al*., 2009a) can be achieved by three nonexclusive mechanisms: (1) by the acquisition of point mutations in the genes encoding either of the two type IIA topoisomerases targeted, DNA gyrase and DNA topoisomerase IV, (2) by reducing the effective concentrations of the drugs in the cytoplasm, either passively by alterations in the membrane permeability or actively by overexpressing efflux systems, and (3) by acquisition of mobile quinolone resistance determinants (Ruiz, 2003; Jacoby, 2005;). While only target modification confers high-level resistance to quinolone antibiotics, the low-level resistance (less than a 10-fold increase in the minimum inhibitory concentration) conferred by the other mechanisms augments the probability of developing such mutations. Mutants that exhibit more than a 100-fold decrease in quinolone sensitivity have been found to carry single point mutations in the chromosomally encoded DNA gyrase subunit gene *gyrA*, with additional enhanced resistance when certain point mutations are simultaneously present in the distantly located *gyrB* gene (Yamagishi *et al*., 1986; Yoshida *et al*., 1988; Cullen *et al*., 1989; Yoshida *et al*., 1990, 1991; Oram & Fisher, 1991; Cambau *et al*., 1993; Ruiz, 2003). Molecular details of these mutations and the binding of quinolones to DNA–topoisomerase complexes have been described elsewhere extensively (Ng *et al*., 1996; Cabral *et al*., 1997; Hiasa & Shea, 2000; Friedman *et al*., 2001; Jacoby, 2005;).

In Gram-negative bacteria, a reduction in antibiotic permeability can be achieved by altering the cell envelope. For example, *E. coli* or *P. aeruginosa* clinical isolates that produce altered lipopolysaccharides differ in the accumulation of quinolones compared with wild-type strains, a nonspecific mechanism thought to involve changes in surface hydrophobicity and consequently affecting passive drug diffusion (Cohen *et al*., 1989; Leying *et al*., 1992; Everett *et al*., 1996; Chenia *et al*., 2006;). However, a reduction in permeability to quinolones is most often achieved by disrupting or downregulating a number of outer-membrane proteins that form channels through which quinolones enter the bacterial cell or by the activation of tripartite multidrug efflux systems belonging to the AcrAB-TolC resistance-nodulation-division (RND) family that can prevent quinolone antibiotics reaching effective concentrations in the cytoplasm (reviewed by Martinez *et al*., 2009b). For instance, *P. aeruginosa* encodes at least 12 RND systems (Stover *et al*., 2000; Schweizer, 2003;), eight of which (MexAB-OprM, MexCD-OprJ, MexEF-OprN, MexXY-OprM, MexJK-OprM, MexHI-OpmD, MexVW-OprM and MexPQ-OpmE) have been reported to export fluoroquinolones and other antibiotics (Chuanchuen *et al*., 2002; Li *et al*., 2003; Sekiya *et al*., 2003; Van Bambeke *et al*., 2003; Mima *et al*., 2005;), although antimicrobial resistance does not appear to be their primary biological function (Aeschlimann, 2003; Van Bambeke *et al*., 2003; Poole, 2004, 2008).

Resistance to quinolone antibiotics can also result from the acquisition of plasmid-borne determinants. The MFS-type efflux pump QepA and the Aac(6′)-Ib-cr enzyme that confers decreased susceptibility to piperazinyl fluoroquinolones such as ciprofloxacin and norfloxacin by acetylation are examples of recently discovered resistance genes carried by plasmids (Robicsek *et al*., 2006; Périchon *et al*., 2007; Yamane *et al*., 2007;). More frequent, however, are the plasmids carrying Qnr quinolone resistance loci, of which at least five families have been identified, mostly in *Enterobacteriaceae* (Jacoby *et al*., 2008; Cavaco *et al*., 2009; Wang *et al*., 2009;). These genes encode proteins of the pentapeptide repeat family that interact with DNA gyrase and topoisomerase IV, preventing quinolone inhibition by mimicking DNA, which probably reduces the availability of holoenzyme–DNA targets for quinolone inhibition (Tran & Jacoby, 2002; Hegde *et al*., 2005; Tran *et al*., 2005a,b;). As with the other quinolone resistance determinants, this function may be considered biologically fortuitous because natural quinolones inhibiting type IIA topoisomerases have not been discovered.

## Quinolones produced by *P. aeruginosa*

Besides Pyo II, which is a 2 : 1 mixture of HQNO and 2-nonyl-4-hydroxyquinoline *N*-oxides (NQNO) (Lightbown, 1950, 1954), with small quantities of 2-undecyl-4-hydroxyquinoline *N*-oxide (UQNO) (Cornforth & James, 1956), *P. aeruginosa* also releases a large number of related molecules. Using ozonolysis and UV absorption spectra in comparison with synthetic standards, Wells *et al*. (1952) identified Pyo Ib as 2-heptyl-4(1*H*)-quinolone (HHQ), Pyo Ic as 2-nonyl-4(1*H*)-quinolone (NHQ) and Pyo III as a monounsaturated alkyl side chain variant of NHQ (Wells, 1952; Wells *et al*., 1952;).

The AQ biosynthetic enzymes of *P. aeruginosa* enable this organism to generate a diverse range of related AQ molecules (Fig. 2 and Box 1). An early study using GC and electron capture MS identified over 20 different AQs (Taylor *et al*., 1995), with HHQ being the most prevalent, followed by NHQ. Variations of these compounds containing saturated and monounsaturated alkyl side chains varying from one to 13 carbons in length, and the two major *N*-oxides, HQNO and NQNO, were also found. Two subsequent studies used electrospray ionization and LCMS to obtain the mass spectra of over 50 different AQs. These mainly consisted of 2-heptyl-3-hydroxy-4(1*H*)-quinolone [termed the *Pseudomonas* quinolone signal (PQS)], HHQ, HQNO and NHQ, with several other saturated and monounsaturated alkyl side chains of various lengths (Lépine *et al*., 2003, 2004). Additional AQs that have been found in significant amounts are 2-nonyl-3-hydroxy-4(1*H*)-quinolone (C9-PQS), 2-undecyl-4-hydroxyquinoline (UHQ), NQNO and UQNO (Taylor *et al*., 1995; Déziel *et al*., 2004; Lépine *et al*., 2004;). Several variations of these compounds are produced (Fig. 2 and Box 1), but many at seemingly biologically insignificant levels, perhaps as a consequence of a lack of specificity of the AQ biosynthetic enzymes for β-keto fatty acids of different chain lengths rather than for any particular biological function. In addition, a metabolite identified as 2,4-dihydroxyquinoline (DHQ) was found in cultures of both *P. aeruginosa* and *Burkholderia thailandensis* (Lépine *et al*., 2007; Zhang *et al*., 2008;). DHQ, although structurally related, is technically not an AQ as it lacks a 2-alkyl chain. It is neither a degradation product nor a precursor of AQs and the precise function of this molecule remains unknown. However, DHQ inhibits the growth and cell viability of mouse lung epithelial MLE-12 cells (Zhang *et al*., 2008) and therefore this molecule may play a role in pathogenicity in respiratory tract infections.

**Box 1. Nomenclature and abbreviations of AQs used in this review**

The structures, IUPAC-based nomenclature and abbreviations of all the major AQs produced by *Pseudomonas aeruginosa* are summarized in Fig. 2. Some non-IUPAC names that have been used to describe some of these same molecules in the scientific literature have been included for clarity. AQs, 2-alkyl-4(1*H*)-quinolones (lactam form) are tautomeric with 2-alkyl-4-hydroxyquinolines (phenolic form), of which the predominance of one form over the other is determined by the pH (Katritzky & Lagowski, 1963; Katritzky *et al*., 1991; Larsen, 2005). For example, it has been demonstrated using p*K*_a_ values for 2-methyl-3-hydroxy-4(1*H*)-quinolone (C1-PQS) that over physiological pH ranges, the neutral 4-quinolone form is the predominant tautomer (Diggle *et al*., 2007). These tautomeric forms are shown in Fig. 2, with their relative ratios indicated by the arrows. Ideally, for consistency and structural accuracy, nomenclature and abbreviations based on only one tautomeric form should have been uniformly adopted. However, this causes some difficulties because in the available scientific literature individual research groups have subjectively referred to these molecules in either one form or the other. For example, even the names used for the two main AQ molecules involved in signalling, PQS and HHQ, are inconsistent with each other with regard to tautomerism: *Pseudomonas* quinolone signal and 2-heptyl-4-hydroxyquinoline. The nomenclature and abbreviations used in this review therefore amount to a compromise between what is technically correct, taking into account IUPAC designations and structural predominance due to physiological pH, and also what has been the prevalent terminology used in the scientific literature for each molecule. Hence, the abbreviation PQS to designate 2-heptyl-3-hydroxy-4(1*H*)-quinolone has been maintained and the alkyl side chain variants of this molecule abbreviated by the number of carbon atoms in the side chain, for example C1-PQS, C9-PQS. The designation HHQ for 2-heptyl-4-hydroxyquinoline has also been maintained, and the other alkyl side-chain derivatives have been abbreviated accordingly (e.g. PHQ for 2-pentyl-4-hydroxyquinoline, etc.). It should be noted that the *N-*oxide series of compounds (AQNOs, e.g. HQNO, 2-heptyl-4-hydroxyquinoline *N-*oxide; NQNO, 2-nonyl-4-hydroxyquinoline *N*-oxide) can adopt the 2-alkyl-1-hydroxy-4(1*H*)-quinolone form (Fig. 2) but not at physiological pH. DHQ or 2,4-dihydroxyquinoline can exist in both, 4-hydroxy-2(1*H*)-quinolone (predominant at physiological pH) and 2-hydroxy-4(1*H*)-quinolone tautomeric forms (Fig. 2), but to avoid confusion and to conform to the literature citations, DHQ is used to denote this molecule in this review. To further help the reader, below is a list of the proposed nomenclature of the AQs that are mentioned along with their abbreviations. Included in this table are the associated synonyms that have been used to describe these same molecules elsewhere in the scientific literature.

**Table:** 

Suggested nomenclature	Synonyms
2-alkyl-4(1*H*)-quinolone (AQ)	2-alkyl-4-hydroxyquinoline (AHQ)
	4-hydroxy-2-alkylquinoline (HAQ)
2-alkyl-4-hydroxyquinoline *N*-oxide (AQNO)	4-hydroxy-2-alkylquinoline *N*-oxide
	2-alkyl-1-hydroxy-4(1*H*)-quinolone
2-heptyl-3-hydroxy-4(1*H*)-quinolone	2-heptyl-3,4-dihydroxyquinoline
*Pseudomonas* quinolone signal (PQS)	2-heptyl-3,4-quinolinediol
3-hydroxy-2-nonyl-4(1*H*)-quinolone (C9-PQS)	3,4-dihydroxy-2-nonylquinoline
	2-nonyl-3,4-quinolinediol
2-pentyl-4-hydroxyquinoline (PHQ)	2-pentyl-4(1*H*)-quinolone
	4-hydroxy-2-pentylquinoline
	2-pentyl-4-quinolinol
2-heptyl-4-hydroxyquinoline (HHQ)	2-heptyl-4(1*H*)-quinolone
	4-hydroxy-2-heptylquinoline
	2-heptyl-4-quinolinol
2-nonyl-4-hydroxyquinoline (NHQ)	2-nonyl-4(1*H*)-quinolone
	4-hydroxy-2-nonylquinoline
	2-nonyl-4-quinolinol
2-undecyl-4-hydroxyquinoline (UHQ)	2-undecyl-4(1*H*)-quinolone
	4-hydroxy-2-undecylquinoline
	2-undecyl-4-quinolinol
2-heptyl-4-hydroxyquinoline *N*-oxide (HQNO)	4-hydroxy-2-heptylquinoline *N*-oxide
	2-heptyl-1-hydroxy-4(1*H*)-quinolone
2-nonyl-4-hydroxyquinoline *N*-oxide (NQNO)	4-hydroxy-2-nonylquinoline *N*-oxide
	2-nonyl-1-hydroxy-4(1*H*)-quinolone
2-undecyl-4-hydroxyquinoline *N*-oxide (UQNO)	4-hydroxy-2-undecylquinoline *N*-oxide
	2-undecyl-1-hydroxy-4(1*H*)-quinolone
2,4-dihydroxyquinoline (DHQ)	4-hydroxy-2(1*H*)-quinolone
	2-hydroxy-4(1*H*)-quinolone
	2,4-quinolinediol

## Properties of AQs

Generally, AQs have a low aqueous solubility. For example, the solubility of PQS is around 1 mg L^−1^ (∼5 μM) in water (Lépine *et al*., 2003). Because of this hydrophobic nature, a high proportion of the AQs are associated with the bacterial outer membrane and with membrane vesicles (MVs) (Mashburn-Warren *et al*., 2008). Of the total amount of PQS produced by *P. aeruginosa* PA14, around 80% appears to be contained within vesicles, in contrast with <1% of either of the *P. aeruginosa N*-acyl-homoserine lactone (AHL)-based signal molecules *N*-(3-oxododecanoyl)-l-homoserine lactone (3-oxo-C12-HSL) and *N*-butanoyl-l-homoserine lactone (C4-HSL) (Mashburn & Whiteley, 2005). The PQS contained within these MVs is seemingly both bioactive and bioavailable because the addition of MVs containing PQS restored the production of pyocyanin in a PQS-negative mutant (PQS being indispensable for the production of pyocyanin in *P. aeruginosa*). The MVs themselves do not seem to have any direct effect on the production of pyocyanin and PQS does not need to be packaged into MVs to exert its effects. MV formation in *P. aeruginosa* PA14 would not seem to be an active process as it occurs independent of growth or of protein synthesis (Mashburn & Whiteley, 2005). Instead, PQS appears to initiate the formation of MVs, into which it is then packaged due to its lipophilic nature (Mashburn & Whiteley, 2005). A mechanism for MV formation has been proposed via the interaction of PQS with the 4′-phosphate and acyl chain of bacterial lipopolysaccharide (Mashburn-Warren *et al*., 2008). Because HHQ is much less efficient in inducing vesicle formation, this activity seems to be dependent on the 3-hydroxy group of PQS and its analogues (Mashburn-Warren *et al*., 2008, 2009). A *pqsH* mutant, deficient in the conversion of HHQ to PQS, is defective in vesicle formation. Of PQS and its analogues, MV formation appears to be optimal when a C7 2-alkyl side chain moiety is present, although C5 and C3 alkyl side chain variants also exhibit some activity and MVs can still be induced to some extent by PQS analogues lacking a 2-alkyl side chain, indicating that this group is dispensable. Additionally, compounds that can inhibit PQS production such as indole and its derivatives reduce MV formation, presumably as there is less PQS available to induce vesicle formation (Tashiro *et al*., 2010). It has been suggested that packaging into MVs could protect PQS from degradation by surrounding cells or competing microbial communities. *Arthrobacter nitroguajacolicus* Rü61 produces the cytoplasmic enzyme Hod [3-hydroxy-2-methyl-4(1*H*)-quinolone 2,4-dioxygenase], which catalyses the 2,4-dioxygenolytic ring cleavage of PQS with the concomitant formation of carbon monoxide and *N*-octanoyl-anthranilic acid (Pustelny *et al*., 2009). As purified Hod is capable of inhibiting AQ signalling when added to cultures of *P. aeruginosa*, at present, the extent of protection conferred by MVs to AQs against enzymatic degradation is unclear.

Rhamnolipids are produced by *P. aeruginosa* and act as biosurfactants, facilitating swarming motility (Caiazza *et al*., 2005). However, rhamnolipids also enhance the aqueous solubility and activity of AQs *in vitro*. The addition of increasing amounts of rhamnolipids enhanced the ability of PQS at a range of concentrations to induce the expression of a *lasB*′-′*lacZ* translational reporter, suggesting that in *P. aeruginosa* the induction of elastase production by PQS is enhanced in the presence of rhamnolipids (Calfee *et al*., 2005). Whether rhamnolipids are indeed effectively utilized to solubilize PQS *in vivo* is, however, not known at present, and with respect to the above reporter system, an excess of rhamnolipids even seems to be detrimental to its expression. A reason for this may be that above a certain threshold concentration, PQS is sequestered into rhamnolipid micelles and therefore becomes less available to the cells (Calfee *et al*., 2005).

AQNOs such as HQNO are potent inhibitors of the cytochrome *bc*_1_ complex and an interesting, but as yet undescribed facet is the mechanism by which *P. aeruginosa* avoids self-poisoning as a consequence of the endogenous production of these molecules. Gram-negative bacteria, as opposed to Gram-positive species such as *Bacillus subtilis* or *S. aureus*, are normally resistant to these compounds, and possible explanations for this have been (1) a reduced cell wall permeability, (2) enzymatic inactivation or (3) an active efflux system to transport the molecule out of the cells (Machan *et al*., 1992). However, none of these mechanisms can account for the resistance of *P. aeruginosa* towards endogenously produced HQNO. Aerobic respiration in *P. aeruginosa* is achieved by a branched electron transport chain ending in five different terminal oxidases, three of which (cytochrome oxidases *cbb*_3_-1, *cbb*_3_-2 and *aa*_3_) receive electrons from ubiquinone via the cytochrome *bc*_1_ complex and cytochromes *c*, whereas the remaining two are the cytochrome *bo*_3_ and the cyanide-insensitive cytochrome *bd* quinol oxidases, which bypass the cytochromes *bc*_1_–*c* electron transfer pathway and get their electrons directly from ubiquinone (Williams *et al*., 2007a). Thus, if the cytochrome *bc*_1_ complexes of *P. aeruginosa* were sensitive to HQNO, this compound alone would have the potential to inhibit three out of five electron transport chains, and in combination with the production of cyanide, 80% of aerobic respiration would be inhibited. *Pseudomonas aeruginosa* is also able to perform anaerobic respiration using nitrogen oxides as terminal electron acceptors. Nitrite, nitric oxide (NOR) and nitrous oxide terminal reductases receive electrons from the cytochrome *bc*_1_ complex, while nitrate reductase (NAR) obtains electrons directly from ubiquinone and also in part via a dedicated membrane-bound formate dehydrogenase (Williams *et al*., 2007a). In this case, HQNO could potentially block all the nitrogen oxide anaerobic respiration, except for the NAR respiratory chain involving formate oxidation. A recent study reported that abolishing AQ production in *P. aeruginosa* enhanced anaerobic growth on nitrate, and that addition of PQS appeared to repress the growth of the wild type and inhibited denitrifying enzymes (Toyofuku *et al*., 2008). Although this effect was attributed to the iron-chelating properties of PQS, the involvement of increased HQNO production cannot be excluded. Whether *P. aeruginosa* prevents self-poisoning with HQNO under aerobic conditions by favouring the cytochrome *bo*_3_ and/or the cyanide-insensitive cytochrome *bd* oxidase pathways or whether there are mechanisms to prevent competitive inhibition of ubiquinone-dependent enzymes under aerobic and anaerobic growth on nitrate remains to be determined.

## Biosynthesis of AQs in *P. aeruginosa*

AQ biosynthesis requires multiple genes, which were initially identified by screening a *P. aeruginosa* transposon mutant library for clones displaying reduced pyocyanin production and were termed *pqsABCDE*, *pqsR* (*mvfR*), *pqsH* and *pqsL* (Cao *et al*., 2001; D'Argenio *et al*., 2002; Gallagher *et al*., 2002; Lépine *et al*., 2002;). The *pqsABCDE* (PA0996-PA1000) genes are arranged in an operon, and adjacent to these are the anthranilate synthase genes *phnAB* (PA1001-PA1002) and *pqsR* (*mvfR*, PA1003). Two other genes are also involved in AQ biosynthesis, *pqsH* (PA2587) and *pqsL* (PA4190), but both of these are located separately elsewhere on the chromosome.

The *pqsR* gene encodes a LysR-type transcriptional regulator that has a helix-turn-helix motif at the *N*-terminus with the first 280 amino acids sharing high similarity (62–71%) with other LysR-type regulators (Cao *et al*., 2001; Maddocks & Oyston, 2008;). PqsR is the transcriptional regulator of both the *pqsABCDE* and the *phnAB* operons and is of crucial importance for AQ production. A mutation in the gene coding for this regulator in *P. aeruginosa* strain PA14 resulted in the abolition of *phnAB* and *pqsABCDE* transcription along with PQS and AQ biosynthesis and had corresponding effects on other virulence determinants including pyocyanin, elastase, exoprotein and 3-oxo-C12-HSL production and consequently the reduced ability to cause disease in plants and animals (Cao *et al*., 2001; Déziel *et al*., 2004;).

The *pqsABCD* genes are involved in the biosynthesis of all AQs (Déziel *et al*., 2004). The first step in this biosynthesis involves the activation of anthranilate by PqsA, an anthranilate coenzyme A ligase (Coleman *et al*., 2008). PqsB and PqsC, which are similar to β-keto-acyl-ACP (acyl carrier protein) synthases involved in fatty acid metabolism, are predicted to elongate acyl side chains of AQ precursors. However, little is currently known about the enzymatic functions of these proteins. PqsD shares some sequence similarity with the Cys-His-Asn active site of the *E. coli* initiation condensing enzyme FabH (Luckner & Ritter, 1965; Ritter & Luckner, 1971; Bredenbruch *et al*., 2005; Zhang *et al*., 2008;). The crystal structure of PqsD with and without a potential covalently bound anthranilate-AQ intermediate product has been resolved recently (Bera *et al*., 2009). Nonpolar mutations in either the *pqsA*, *pqsB* or *pqsD* genes completely abolish the production of AQs (Diggle *et al*., 2003; Zhang *et al*., 2008;).

In addition to these four AQ biosynthesis genes, the *pqsABCDE* operon also encodes PqsE, which has sequence similarities to proteins of the metallo-β-hydrolase superfamily. This extensive family of hydrolytic enzymes mediates a wide range of functions, such as β-lactamases, glyoxalases, AHL-lactonases and arylsulphatases. These enzymes are usually characterized by a conserved metal ion-binding HXHXDH amino-acid motif, which is also found in PqsE and that forms an active site able to bind two iron atoms (Yu *et al*., 2009). However, although the PqsE the crystal structure is available, little knowledge has been gained about its exact function, its natural substrate remaining unknown. The deletion of *pqsE* reduces the production of several virulence factors including pyocyanin, lectin and hydrogen cyanide (HCN), while overexpression of *pqsE* has the opposite effect. A transcriptomic analysis has recently revealed that the abundances in the mRNAs of ≈400 genes depend on the level of expression of *pqsE*, and that virulence in plant and animal infection models in the absence of AQ depends on this gene (Rampioni *et al*., 2010). The activity of PqsE has also been reported to be dependent on RhlR, which acts downstream, but in synergy with PqsE (Hazan *et al*., 2010). Interestingly, PqsE is not involved in AQ biosynthesis, and appears to be a crucial element mediating the cellular response to PQS to achieve full virulence (Gallagher *et al*., 2002; Diggle *et al*., 2003; Déziel *et al*., 2005; Farrow *et al*., 2008;).

The *pqsH* gene encodes a predicted FAD-dependent monooxygenase that hydroxylates the 3′ carbon atom of HHQ in the final step of PQS biosynthesis. As such, *pqsH* mutants do not produce 3-hydroxylated quinolones, but continue to produce other AQs (Déziel *et al*., 2004). Because *pqsH* is regulated by LasR, but not by PqsR, it is therefore conceivable that under certain circumstances, a differential regulation of these two elements may lead to the overproduction of HHQ with respect to PQS, due to a lack of PqsH (Déziel *et al*., 2004).

The *pqsL* gene encodes a second, distinct monooxygenase that is required for the synthesis of HQNO and related *N*-oxides via oxidation of the quinolone ring nitrogen atom. The detailed mechanism of AQNO biosynthesis is still unknown, but interestingly, HHQ does not appear to be a precursor of HQNO, as the addition of deuterated HHQ to a culture of *P. aeruginosa* resulted in the biosynthesis of deuterated PQS, but not of deuterated HQNO (Déziel *et al*., 2004). Additionally, a *pqsL* mutant overproduces PQS compared with its isogenic wild-type parent, suggesting that PqsL interacts with and diverts a fraction of the HHQ precursor products towards AQNO biosynthesis and away from HHQ and PQS biosynthesis (D'Argenio *et al*., 2002).

**Fig. 4:**
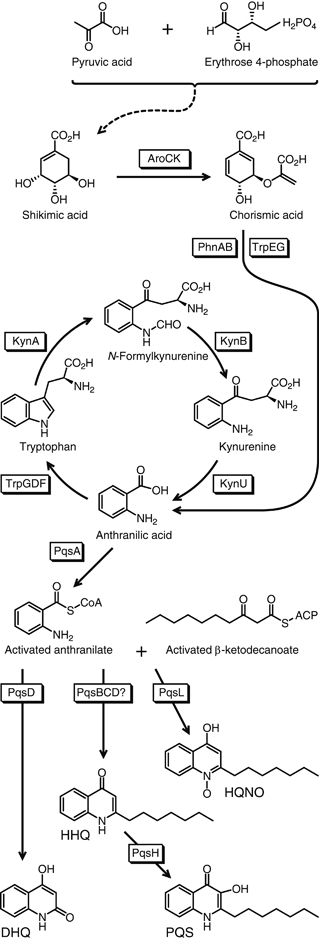
Proposed biosynthetic pathway of PQS, HHQ, HQNO and DHQ in *Pseudomonas aeruginosa*. AQs are derived from a condensation reaction between anthranilate and β-keto fatty acids. Anthranilate is derived from either the PhnAB/TrpEG or the KynABU metabolic pathways using either chorismate or tryptophan as precursors, respectively. Anthranilate is first activated with coenzyme A (CoA) by PqsA. Anthranilate-CoA and an activated β-ketodecanoate are condensed, possibly via the PqsBCD enzymes to HHQ, releasing CO_2_ and H_2_O. The monooxygenase PqsH converts HHQ to PQS. HQNO is derived from the same starting products as HHQ, but utilizes the additional monooxygenase PqsL. HHQ is not a precursor for HQNO. DHQ, which technically is not an AQ, is produced by PqsD independent of PqsB and PqsC.

A simplified scheme for the biosynthesis of AQs is detailed in Fig. 4. Before the role of these molecules in signalling was discovered, an AQ biosynthesis pathway had already been proposed. This was based on radiolabelled precursor feeding experiments, which indicated condensation of anthranilic acid with β-keto fatty acids, releasing CO_2_ and H_2_O (Cornforth & James, 1956; Luckner & Ritter, 1965; Ritter & Luckner, 1971;). This was confirmed more recently by MS and nuclear magnetic resonance (NMR) analysis of the AQs produced after feeding ^13^C and ^15^N isotope-labelled precursors to *P. aeruginosa* (Bredenbruch *et al*., 2005). This study also ruled out a second possible pathway of AQ biosynthesis that involved the formation of a kynurenic acid precursor resulting from a reaction between orotic acid and anthranilate. The heterologous expression of AQ biosynthesis genes in *E. coli* revealed that the production of DHQ (Fig. 2) only requires PqsA and PqsD (Zhang *et al*., 2008). Activated anthraniloyl-CoA, generated by PqsA, is transferred to the cysteine residue in the active site of PqsD (unactivated anthranilate does not transfer). Here, it reacts with either malonyl-CoA or malonyl-ACP to form 3-(2-aminophenyl)-3-oxopropanoyl-CoA, a short-lived intermediate that undergoes an internal rearrangement to form DHQ. Some variation of this biosynthesis, utilizing longer chain β-keto fatty acids in place of malonyl-CoA or malonyl-ACP, is possibly the mechanism by which AQ molecules such as HHQ are produced. However, because the above process only utilizes PqsA and PqsD, AQ biosynthesis is likely to be more complex as the functions of PqsB and PqsC are still unclear (Zhang *et al*., 2008; Bera *et al*., 2009;).

The substrates required for AQ biosynthesis have also been investigated. Two pairs of genes responsible for anthranilate biosynthesis had been described previously: *phnAB* (Essar *et al*., 1990a), located adjacent to the *pqs* operon (Gallagher *et al*., 2002), and *trpEG*, which encode enzymes involved in tryptophan biosynthesis (Essar *et al*., 1990b). The *phnAB* genes code for proteins resembling the *E. coli* anthranilate synthase subunits TrpE and TrpG (Essar *et al*., 1990a) and are cotranscribed by PqsR (Cao *et al*., 2001). It was initially thought that these would provide anthranilate as a precursor for phenazine biosynthesis as inactivation of these genes reduced pyocyanin production. However, it was subsequently shown that PhnA and PhnB do not appear to be involved in this function (Mavrodi *et al*., 2001) and therefore the reduction in pyocyanin production in a *phnAB* mutant is instead most likely to be the consequence of the reduced availability of AQs. In addition to PhnA and PhnB, TrpE and TrpG also direct the synthesis of anthranilate from chorismate, which can then be utilized either for tryptophan or for AQ biosynthesis. A third source of anthranilate comes from the homologues of the tryptophan 2,3-dioxygenase KynA, the kynurenine formamidase KynB and the kynureninase KynU to produce anthranilate from tryptophan (Kurnasov *et al*., 2003; Farrow & Pesci, 2007;). Detailed analyses indicate that in rich growth media containing the aromatic amino acid tryptophan, the kynurenine pathway is the main source of anthranilate for AQ production, whereas the *phnAB* genes supply anthranilate in minimal media in the absence of exogenous tryptophan (Farrow & Pesci, 2007). Interestingly, increased PQS production by *P. aeruginosa* strains isolated from infected CF lungs has been correlated with the presence of aromatic amino acids in the growth medium (Palmer *et al*., 2005). In this case, the transcription of *pqsA* was found to be induced by tryptophan, phenylalanine and tyrosine, while the nonaromatic amino acid serine had little effect. The kynurenine pathway may therefore be the principal source of anthranilate in a lung infection context.

The requirement of anthranilate for PQS production has been demonstrated. When *P. aeruginosa* PAO1 parent and isogenic *las* QS mutants unable to produce PQS were grown in the presence of anthranilate labelled with ^14^C in the heteroaromatic ring, most of the radioactivity was found in the AQ extracts for those strains able to generate PQS, whereas very little was found in the supernatant extracts of the QS mutants (Calfee *et al*., 2001). This suggested that the strains not producing PQS would incorporate anthranilate, but not convert it into AQs. Additionally, when *P. aeruginosa* was grown with increasing amounts of methyl-anthranilate, PQS biosynthesis levels were reduced as this compound acted as a competitor of anthranilate (Calfee *et al*., 2001). The production of elastase, which is dependent on PQS signalling, was also inhibited by methyl-anthranilate in a concentration-dependent manner. At 1.5 mM, methyl-anthranilate practically abolished elastase production, the suggested consequence of a much reduced level of PQS production.

Feeding experiments with isotope-labelled AQ precursors such as ^15^N-anthranilate coupled with GC–MS analysis resulted in the production of AQs having incorporated around 66% of ^15^N, further demonstrating that anthranilate serves as a common precursor for AQs and that the heteroaromatic nitrogen in the quinolone ring originates from this molecule (Bredenbruch *et al*., 2005). Similarly, feeding labelled ^13^C-acetate to *P. aeruginosa* PAO1 demonstrated that the heteroaromatic ring of the quinolone moiety was formed from acetate. The resulting GC–MS fragmentation pattern, together with confirmation by NMR spectroscopy, indicated that the mechanism of this reaction was via a direct head-to-head reaction involving anthranilate and β-keto fatty acids derived from acetate (Bredenbruch *et al*., 2005). β-Keto fatty acids are therefore essential precursors in the biosynthesis of AQs. Some studies had suggested that there is a link between rhamnolipid biosynthesis and AQ production, which was interesting because rhamnolipids are composed of a rhamnose moiety and fatty acids of the same chain lengths as those involved in AQ biosynthesis. Rhamnolipids have also been shown to increase PQS solubility and may mediate this function *in vivo* (Calfee *et al*., 2005). It was initially thought that *rhlG* coded a potential β-ketoacyl-ACP reductase that could participate in the provision of fatty acids utilized as a substrate for AQ biosynthesis (Bredenbruch *et al*., 2005) as RhlG was assumed to direct the incorporation of these fatty acids into rhamnolipids (Campos-García *et al*., 1998; Déziel *et al*., 2003; Soberón-Chávez *et al*., 2005;). However, recent studies have contradicted this, as an *rhlG* mutant was unaltered in rhamnolipid production compared with the corresponding wild type (Zhu & Rock, 2008). Furthermore, the crystal structure of RhlG revealed that its function was inconsistent with the proposed fatty acid biosynthetic pathway (Miller *et al*., 2006). Therefore, it appears that RhlG is not involved in rhamnolipid or AQ biosynthesis.

In *P. aeruginosa*, PQS is likely to be the end product of the AQ synthetic pathway or is not substantially converted into other molecules, as when labelled PQS was added to wild-type cultures, no additional compounds could be identified (Déziel *et al*., 2004).

## Quorum sensing (QS) and AQ production in *P. aeruginosa*

When favourable nutritional conditions are encountered, bacteria will proliferate to form established multicellular communities that have the potential to adapt to and modify their environment. This allows further exploitation of nutrient resources that would otherwise be restricted for individual cells. The mechanism by which a bacterium adapts from the lifestyle of an individual cell to a community capable of modifying their environment has been termed QS and it is defined as a mechanism by which bacteria regulate specific target genes in response to a critical concentration of endogenously produced signal molecules dedicated to the probing of the cell population density (Venturi, 2006; Williams *et al*., 2007b;). This process is mediated by the production and sensing of autoinducers, small signalling molecules, whose concentration in the extracellular medium reflects cell population density. *Pseudomonas aeruginosa* produces two AHLs as QS signal molecules, each acting as the autoinducer of a specific sensing and responding system: 3-oxo-C12-HSL acts on the *las* system and C4-HSL acts on the *rhl* system. The core of each system is composed of a synthase producing an AHL for the activation of a specific transcriptional regulator: LasI produces 3-oxo-C12-HSL for the activation of LasR (Gambello & Iglewski, 1991; Passador *et al*., 1993; Pearson *et al*., 1994;) and RhlI produces C4-HSL for the activation of RhlR (Latifi *et al*., 1995; Pearson *et al*., 1995; Winson *et al*., 1995;). Initially, each synthase gene is expressed at basal levels and the AHLs produced diffuse into the surrounding medium. Autoinduction is achieved when the accumulation of an AHL reaches a threshold concentration and the activated transcriptional regulators LasR and RhlR further enhance the expression of the synthase genes *lasI* and *rhlI*, respectively, generating positive feedback loops (Seed *et al*., 1995). When the transcriptional regulators are activated they will induce the transcription of overlapping subsets of genes. For example, LasR will induce the production of virulence factors such as elastase (Passador *et al*., 1993) and pyoverdin (Stintzi *et al*., 1998), while RhlR will increase the production of rhamnolipid biosurfactants (Ochsner & Reiser, 1995), cytotoxic lectins, pyocyanin and elastase, among other virulence factors (Pearson *et al*., 1997). In addition to some overlap between the genes targeted by both AHL QS systems due to the similarities of the palindromic *las*/*rhl* boxes recognized by LasR and RhlR (Schuster *et al*., 2004; Schuster & Greenberg, 2006, 2007), activated LasR will also induce the *rhl* system (Latifi *et al*., 1996), creating a hierarchical regulatory network, which in turn is further modulated by additional regulatory elements (reviewed in von Bodman *et al*., 2008 and in Williams & Cámara, 2009).

Besides the AHL-based QS systems, *P. aeruginosa* utilizes an autoinducer regulatory system based on the AQs. This system relies on the PQS and its precursor molecule HHQ to control global gene expression (Pesci *et al*., 1999; Déziel *et al*., 2004;). The transcriptional regulator PqsR controls the expression of the *pqsABCDE* and *phnAB* biosynthetic operons and therefore *pqsR* is essential for the production of AQs (Gallagher *et al*., 2002; Déziel *et al*., 2004; McGrath *et al*., 2004;).

The *pqsR* gene is convergently transcribed with respect to the *pqsABCDE*-*phnAB* operons and two transcriptional start sites have been mapped 190 and 278 bp upstream of its start codon (Wade *et al*., 2005). The distant promoter appears to have a typical σ^70^-binding site signature, indicative of basal transcription, and a putative *las*/*rhl* box operator sequence is found centred 239–258 bp upstream of this transcriptional start site (517–536 bp upstream of the start codon). *In vitro*, PqsR binds at two different locations upstream of *pqsA*, and the strength and position of the binding depend on the presence of PQS (Wade *et al*., 2005). The *pqsA* transcriptional starting point has been mapped 71 bp upstream of the start codon (McGrath *et al*., 2004). Alterations of a LysR-type box located at −45 in the *pqsA* promoter can result in the loss of PqsR-binding capacity and in the reduction of transcription initiation, suggesting that this element plays a central role in the regulation of the *pqsABCDE* operon by PqsR and PQS (Xiao *et al*., 2006b). Overexpression of *pqsR* strongly repressed the transcription of *antA*, which encodes an anthranilate 1,2-dioxygenase. This is thought to ensure an adequate supply of anthranilate for the biosynthesis of AQs by reducing its metabolic degradation (Oglesby *et al*., 2008).

When the *pqsABCDE* operon and *pqsR* were cloned in *E. coli* and expressed from their native promoters, HHQ and NHQ were produced, but not PQS because *E. coli* lacks a *pqsH* homologue. Similarly, compared with the wild type, the activity of the *pqsA* promoter and AQ production levels (except for PQS) remained comparable when *pqsH* was disrupted. This indicates that in addition to PQS, other AQs can also act as autoinducers (Xiao *et al*., 2006a). It has been suggested that HHQ induces a conformational change in PqsR, as binding of PqsR to the *pqsA* promoter *in vitro* is enhanced by HHQ, although not as much as with PQS. In an AQ-negative double *pqsA pqsH* mutant derived from strains PAO1 or PA14, PQS was found to be 100 times more potent at inducing the *pqsA* promoter than HHQ (Xiao *et al*., 2006a; Diggle *et al*., 2007;). In strain PA14, the deletion of *pqsH* reduced the overall expression of the *pqsR* regulome by less than twofold, and the addition of exogenous PQS to this mutant did not revert the expression levels of this regulome substantially above wild-type levels, further implying a role for HHQ in inducing many of the genes. An exception to this was *phzA1*, as PQS appears to be essential for the transcription of this gene and for the production of pyocyanin (Xiao *et al*., 2006a). Altogether, these studies indicate that HHQ acts as an autoinducer independent of PQS. Other AQs such as NHQ can also activate PqsR and as such could potentially be considered as autoinducers, although not as potent as PQS (Xiao *et al*., 2006a; Fletcher *et al*., 2007;).

**Fig. 5:**
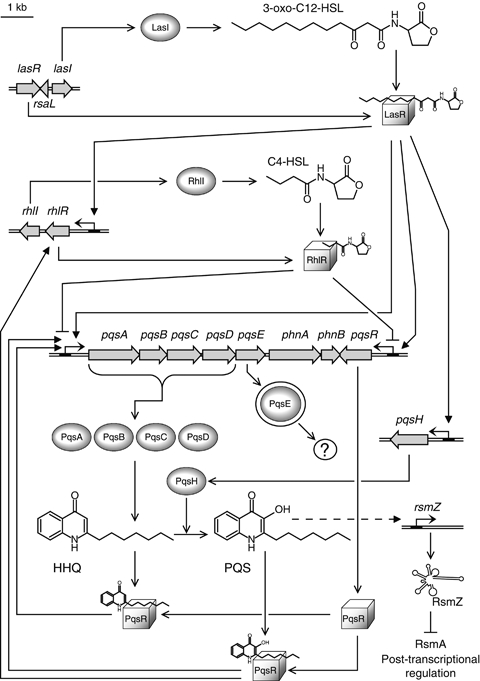
Regulation of AQ production in*Pseudomonas aeruginosa*. The *las* QS system positively regulates the transcription of *pqsR*, *pqsABCDE* and *pqsH*. The PqsABCD proteins synthesize HHQ, which is converted to PQS by PqsH. Autoinduction occurs when either HHQ or PQS binds to PqsR and enhances the expression of the *pqs* operon. The *rhl* QS system, also positively controlled by the *las* system, exerts a negative effect on the AQ system, although it is itself positively regulated by AQs. The terminal output of this regulatory network is the PqsE protein of still unknown enzymatic function. In addition, PQS, via an unknown mechanism, positively controls the transcription of the small RNA *RsmZ*, which in turns has a negative effect on the RNA-binding protein RsmA involved in post-transcriptional regulation. Biosynthetic enzymes are represented by globular shapes, while transcriptional regulators are shown as cubes. Filled arrows and blunted lines represent positive and negative regulation, respectively.

The *las* and *rhl* QS systems are linked to AQ production and regulation, forming an incoherent feed-forward loop likely to produce accelerated pulse-like responses (Alon, 2007): the *las* system positively controls AQ production by inducing the *pqsR* and *pqsA* promoters and the *rhl* system downregulates its effects (Pesci *et al*., 1999; McKnight *et al*., 2000; McGrath *et al*., 2004; Wade *et al*., 2005; Xiao *et al*., 2006b;) (Fig. 5). In a *lasR* mutant, transcription of *pqsR* is reduced about fourfold compared with the wild type (Wade *et al*., 2005) and LasR appears to induce *pqsR* transcription by binding to a conserved *las*/*rhl* box situated 517–536 bp upstream of its translational start site (McGrath *et al*., 2004; Xiao *et al*., 2006b; Gilbert *et al*., 2009;). In line with this, a transcriptional *pqsR*-*lacZ* fusion can be significantly induced in *E. coli* expressing *lasR* by the addition of 3-oxo-C12-HSL, indicating that the LasR/3-oxo-C12-HSL system acts as an inducer of *pqsR* (Wade *et al*., 2005). A *lasR* mutant accumulates the HHQ series of AQs, but produces very little PQS early in growth, a consequence of LasR also positively controlling the expression of *pqsH*, which encodes the monooxygenase required for the conversion of HHQ to PQS (Whiteley *et al*., 1999; Gallagher *et al*., 2002; Déziel *et al*., 2004;). The transcription of *pqsA* is considerably reduced in a *lasI* mutant (McGrath *et al*., 2004). However, a functional *las* QS system is not required for AQ biosynthesis, as a *lasR* mutant still produces PQS in the late stationary phase and expressions of *pqsR* and *pqsH* in a *lasR* mutant are delayed, but not abolished during growth (Diggle *et al*., 2003; Xiao *et al*., 2006b;). As *rhlR* overexpressed from a plasmid partially overcomes the delay in PQS production caused by a *lasR* mutation in strain PA14 (Dekimpe & Déziel, 2009), it appears that RhlR could replace some of the functions of LasR with respect to the *pqsA* and *pqsH* promoters to induce the production of PQS, although this is somewhat paradoxical because RhlR is generally considered to be a repressor of AQ production and indicates that the current LasR-RhlR-AQ QS hierarchy model in *P. aeruginosa* may be somewhat more sophisticated than currently thought.

While the *las* QS system positively regulates AQ and PQS production, the *rhl* system acts as a negative modulator of their regulatory effects (Fig. 5). A 50% increase in *pqsR* transcription has been observed in an *rhlR* mutant, suggesting in this case that RhlR has a repressive effect (Wade *et al*., 2005). Similarly, transcription from the *pqsA* promoter is enhanced in an *rhlI* mutant and addition of C4-HSL to antagonize the induction of *pqsA* by 3-oxo-C12-HSL, with the consequence of reducing the production of PQS (McGrath *et al*., 2004). Two *las*/*rhl* boxes are found at 311 and 151 bp upstream of the *pqsA* transcriptional start site (Xiao *et al*., 2006b). Deletion of the distal *las*/*rhl* box in this promoter increases transcription, while additional deletion of the proximal box does not further increase *pqsA* promoter activity. The deletion of *rhlR* causes an increase in the transcription of *pqsA* independent of the presence of the −311 box, suggesting that RhlR binds to this box and causes a downregulation of the *pqsA* promoter, whose mechanism is still unclear. *In vitro* electrophoretic mobility shift assays carried out on a 253-bp DNA fragment containing part of the *pqsA* promoter using lysates of *E. coli* producing RhlR in the presence or absence of C4-HSL-RhlR did not indicate binding to this region; however, the fragment used did not include the *las*/*rhl* box situated 311 bp upstream of the transcriptional starting point (Wade *et al*., 2005). Identification of LasR targets *in vivo* using chromatin immunoprecipitation coupled to DNA microarray hybridization (ChIP-chip) identified this distal *las*/*rhl* box as a LasR-binding site (Gilbert *et al*., 2009). As the *rhl* system is itself driven by the production of PQS, a negative autoregulatory feedback loop is formed (Diggle *et al*., 2003). The simultaneous provision of exogenous C4-HSL and PQS restores *rhlI* transcription levels in a *lasR* mutant comparable with the wild type. However, under the same conditions, the addition of these molecules separately did not cause increased *rhlI* transcription, suggesting a synergistic mechanism involving the two signalling molecules (McKnight *et al*., 2000).

Thus, in *P. aeruginosa*, the autoinducible AQ system is upregulated by the *las* and downregulated by the *rhl* QS systems. AQ production is furthermore indirectly self-limited by the positive regulatory effects it exerts on the *rhl* QS system (Fig. 5).

## Regulation of virulence factor expression by AQs

The first demonstration that AQs regulate virulence factor production in *P. aeruginosa* was that PQS positively controlled the expression of the *lasB* (elastase) gene (Pesci *et al*., 1999). It was later shown that this effect was considerably enhanced when PQS and C4-HSL acted synergistically to upregulate *lasB* expression (McKnight *et al*., 2000). The regulation of virulence factor production by AQs is not restricted to elastase. Addition of PQS upregulates the expression of *lecA* and pyocyanin production in a concentration-dependent manner (Diggle *et al*., 2003). However, PAO1 cultures growing in the presence of PQS above a concentration of 100 μM had an extended lag phase and reached reduced ODs at the stationary phase. Despite this, the expression of *lecA* occurred at lower population densities and therefore the maximal expression was still observed during the early stationary phase, although the addition of PQS still resulted in an advancement of *lecA* expression and elastase and pyocyanin production into the logarithmic phase (Diggle *et al*., 2003). It worth noting that these effects were not seen when either HHQ or 3-formyl-HHQ instead of PQS was added to the cultures. Previous studies found that both RhlR and RpoS are essential for *lecA* expression (Winzer *et al*., 2000) and addition of PQS failed to restore *lecA* transcription in *rhlR* or *rpoS* mutants, confirming the importance of these two regulators for *lecA* promoter activity. However, PQS was able to overcome the repression of *lecA* by the H-NS-type protein MvaT and the post-transcriptional regulator RsmA (Diggle *et al*., 2003). We have recently found that PQS, but not HHQ, induces the transcription of the small regulatory RNA RsmZ (Fig. 5), a mechanism that explains how post-transcriptional regulation by RsmA can be overcome by PQS and reveals that this molecule can act on the expression of virulence genes at both the transcriptional and the post-transcriptional levels (S. Heeb *et al*., unpublished data).

Virulence factor production is also affected when AQ production is inhibited. Addition of the anthranilate analogue, methyl-anthranilate, to *P. aeruginosa* caused a decrease in the production of PQS and a subsequent reduction in elastase produced (Calfee *et al*., 2001). The effects observed with methyl-anthranilate are not restricted to elastase, with concentrations of 500 μM completely inhibiting the expression of *lecA* and pyocyanin production, but with no adverse effect on growth. This effect could partially be restored by the provision of exogenous PQS (Diggle *et al*., 2003).

The subset of genes regulated by AQs has now been examined in greater detail using transcriptomic analysis. It has been found that PqsR, through the induction of the *pqsABCDE* operon and the action of PqsE, positively regulates a subset of LasR- and RhlR-dependent genes. A *pqsR* mutant of strain PA14 displayed the upregulation of 121 and the repression of 22 mRNAs when compared with the corresponding wild type (Déziel *et al*., 2005). In this *pqsR* mutant, the transcription of the *pqsABCDE* and *phnAB* operons was abolished and that of *pqsR* itself was reduced. The transcription of the *phz1* operon, *hcnABC*, *chiC* (chitinase), *mexGHI*-*opmD*, *lecA* and *lecB* was also found to be reduced in the absence of PqsR.

However, the role of AQs in the regulation of virulence gene expression is now the subject of some debate. It had been demonstrated previously that in both *pqsR* and *pqsE* mutants, pyocyanin production, *phzA1* expression, LecA, elastase and rhamnolipid production levels were considerably reduced compared with the wild type (Cao *et al*., 2001; Gallagher *et al*., 2002; Diggle *et al*., 2003; Déziel *et al*., 2005;) and that the addition of PQS, HHQ or HQNO to these mutants could not restore these phenotypes (Gallagher *et al*., 2002; Diggle *et al*., 2003; Déziel *et al*., 2005;). Altogether, these studies suggested that AQ production may not be indispensable for the regulation of these phenotypes. A subsequent study shed new light on the mechanisms by which AQs induce gene transcription by revealing that PqsE alone can drive the expression of the target genes, through the *rhl* QS system (Farrow *et al*., 2008). By expressing PqsE in AQ-negative *pqsA* or *pqsR* mutants, it was demonstrated that pyocyanin, rhamnolipid and elastase production could be restored in the absence of AQs. This restoration of exoproducts was not observed in an *rhlR* mutant, which suggests that PqsE may exert its effects through the *rhl* system (Farrow *et al*., 2008). These findings raise a number of intriguing questions as to the function of AQs in *P. aeruginosa*. For example, is the primary function of both, PQS and HHQ, to bind PqsR and to upregulate the *pqsABCDE* operon, thereby forming, on the one hand, an autoinduction loop and ultimately, on the other, producing as the major output, an increase in the levels of PqsE? Another intriguing question raised by the data is about the function of PQS itself. There are conflicting reports on the necessity of PQS for virulence in different *P. aeruginosa* wild-type strains, although different hosts have been used: PQS has been shown to be necessary for the virulence of strain PAO1 in nematodes (Gallagher *et al*., 2002), but unnecessary for PA14 in a burned mouse model (Xiao *et al*., 2006a). There are also conflicting reports as to the efficacy of PQS at inducing the *pqsA* promoter via PqsR. One study found that in PA14, PQS was more effective than HHQ at upregulating *pqsA* (Xiao *et al*., 2006a), but conversely, another study demonstrated that in strain PAO1, PQS was the less effective molecule (Fletcher *et al*., 2007). This contradiction may be due to differences in strain-specific mechanisms, but taken together with new research on the role of PqsE, PQS may not be as important to the direct regulation of virulence factors in *P. aeruginosa* as first envisioned and may have evolved as a fortuitous byproduct with other functions (Bredenbruch *et al*., 2006; Diggle *et al*., 2007;). Furthermore, the primary role of PqsR requires some further clarification. It is probable that the loss of virulence noted in *pqsR* mutants (Cao *et al*., 2001; Déziel *et al*., 2005;) is primarily due to the corresponding loss of PqsE production, seen in the fact that mice mortality in strain PA14 was much decreased from the wild type and was equivalent in both *pqsA* and *pqsE* mutants (Déziel *et al*., 2005). Therefore, the primary role of PqsR may be that it is responsible for the expression of *pqsE* via the production of AQs and the corresponding autoinduction of the *pqsABCDE* operon, at least as far as the production of pyocyanin and expression of *lecA* are concerned. The induction of pyocyanin production by the AQ QS system further leads to the regulation of the PYO stimulon, a set of around 50 genes whose expression is affected, primarily via the transcriptional regulator SoxR, by this phenazine (Dietrich *et al*., 2006).

## Role of AQs in iron metabolism

In addition to its role as a cell-to-cell signalling molecule, PQS is also able to chelate ferric iron (Fe^3+^). The presence of the 3′-hydroxy group on the molecule mediates this and allows two or three PQS molecules to bind Fe^3+^ at physiological pH ranges of 6–8. Compounds similar to PQS (such as C9-PQS) also possess iron-binding capabilities, but molecules lacking the 3-hydroxy group such as HHQ are unable to do so (Bredenbruch *et al*., 2005; Diggle *et al*., 2007;).

Addition of PQS to *P. aeruginosa* cultures upregulates the genes involved in the production of the siderophores pyoverdine and pyochelin, which are produced in response to iron starvation, as indicated by the upregulation of siderophore-mediated iron transport systems such as the pyochelin biosynthetic clusters (*pchDCBA* and *pchEGF*), the iron pyochelin outer-membrane receptor *fptA* and the pyoverdine genes *pvdE* and *pvdS* (Bredenbruch *et al*., 2005; Diggle *et al*., 2007;). The *pch* genes were upregulated at 5, 11 and 20 h after inoculation between 3- and 25-fold. Also, the genes *pvdJAD* encoding pyoverdine synthetases were upregulated between 2- and 10-fold at 11 and 20 h (Bredenbruch *et al*., 2005). Quantitative real-time PCR showed that *pvdA* and *pchE* are upregulated by 6- and 17-fold, respectively, upon addition of 20 μM PQS (Diggle *et al*., 2007). Also, in strain PAO1 wild type as well as in *pqsA*, *pqsE* or *pqsR* mutants, the addition of PQS, but not of HHQ, strongly induced pyoverdine production (Diggle *et al*., 2007).

PQS, with its effect on free iron levels, also affects the transcription of other genes. During growth in iron-replete media, both *lecA* and *pqsA* were strongly induced by the addition of 50 μM PQS in a PAO1 *pqsA* mutant. However, the induction of *pqsA* was not due to the iron-chelating properties of PQS because when grown in an iron-deficient casamino acid (CAA) medium, PQS, PQS–Fe^3+^ (3 : 1) and HHQ all induced the *pqsA* promoter, but methyl-PQS did not (Diggle *et al*., 2007).

Around 60% of the PQS produced by *P. aeruginosa* is associated with the cell envelope (Lépine *et al*., 2003; Diggle *et al*., 2007;), and the membranes of cells grown in iron-rich media are visibly pink due to complexed Fe^3+^, possibly stored in AQ-containing inclusion bodies (Royt *et al*., 2001, 2007). Therefore, there is the possibility that PQS could act as an iron trap and storage molecule in the cell membrane and that it may be able to deliver iron directly to the cells. However, experiments carried out with a *P. aeruginosa pvdD*/*pchEF* double mutant, which lacks any iron acquisition systems, revealed that it was unable to grow in an iron-deficient CAA medium in the presence of added PQS. In contrast, this mutant had a similar growth compared with the parental PAO1 strain when exogenous PQS was not added to the medium. These data suggest that although PQS may trap iron in the cell membrane, it is unlikely that it can act as a siderophore *per se* (Diggle *et al*., 2007).

Iron-dependent regulation of AQ production appears to be controlled by the availability of one of their precursors, anthranilate. Under iron-limiting conditions, the ferric uptake regulator Fur does not repress the transcription of two genes *prrF1* and *prrF2*, encoding small regulatory RNAs (Wilderman *et al*., 2004), which post-transcriptionally repress the expression of the *antABC* and *catBCA* operons specifying enzymes for the degradation of anthranilate. Hence, in a *prrF1 prrF2* double mutant, PQS production is abolished under iron-limiting conditions, probably as a consequence of anthranilate depletion (Oglesby *et al*., 2008). Therefore, under iron-limiting conditions, the supply of anthranilate for the biosynthesis of AQs is controlled by Fur and the PrrF sRNAs, an effect that was further reinforced by the iron starvation response resulting from the iron-chelating property of PQS (Bredenbruch *et al*., 2006; Diggle *et al*., 2007;).

Because HHQ performs functions similar to those of PQS, such as the induction of the *pqsA* promoter (Xiao *et al*., 2006a; Diggle *et al*., 2007;), many of the specific effects observed upon addition of PQS may be due to its iron-chelating properties. It is also probable that the production of PQS and its chelating effects could confer a survival advantage when *P. aeruginosa* is growing with other competing microorganisms in iron-limited environments. The red-coloured PQS–Fe^3+^ complex can also be toxic to other organisms. For example, its production has been found to confer the ‘red death’ lethal phenotype to *P. aeruginosa* in a *Caenorhabditis elegans* infection model (Zaborin *et al*., 2009).

Iron availability also influences the levels at which AQs induce the activity of PqsR as a transcriptional activator, and therefore, iron also acts directly as a modulator of the AQ signalling system in *P. aeruginosa* (Hazan *et al*., 2010). Interestingly, iron has also been found bound to PqsE, although without knowledge of the function of this enzyme, the biological significance of this remains unclear (Yu *et al*., 2009).

## Additional regulators of AQ production in *P. aeruginosa*

Besides autoinduction by PQS and its precursor HHQ, modulation by the *las* and *rhl* QS systems, and metabolic and regulatory adjustments following iron availability, AQ production is regulated by additional factors (Table 1). For example, AQ production is enhanced under phosphate-limiting conditions. In *P. aeruginosa*, the transcriptional regulator PhoB mediates responses to phosphate limitation (Anba *et al*., 1990). As a PHO box has been found overlapping the distal transcriptional starting point of *pqsR* and as AQ production is no longer enhanced in a *phoB* mutant, these elements may mediate the increased AQ production observed following phosphate limitation (Jensen *et al*., 2006).

**Table 1:** Factors influencing AQ production

Factors	Mechanisms	References
PqsR and AQs	Some AQs positively regulate AQ biosynthesis by autoinduction. PqsR binding to the *pqsA* promoter is enhanced and the transcription of the *pqsABCDE* operon is induced by PQS, and to different levels by other AQs such as HHQ and NHQ	Pesci *et al*. (1999), Gallagher *et al*. (2002), Déziel *et al*. (2004), McGrath *et al*. (2004), Wade *et al*. (2005), Xiao *et al*. (2006a,b);, Diggle *et al*. (2007), Fletcher *et al*. (2007), Oglesby *et al*. (2008)
*las* QS system	LasR and 3-oxo-C12-HSL positively regulate AQ production by inducing the transcription of *pqsR* and *pqsABCDE*, and further enhances the production of PQS by inducing *pqsH*	Pesci *et al*. (1999), Whiteley *et al*. (1999), McKnight *et al*. (2000), Gallagher *et al*. (2002), Diggle *et al*. (2003), Déziel *et al*. (2004), McGrath *et al*. (2004), Wade *et al*. (2005), Xiao *et al*. (2006a,b);, Dekimpe & Déziel (2009), Gilbert *et al*. (2009)
*rhl* QS system	RhlR negatively regulates AQ production by repressing *pqsR* transcription. The expression of *rhlI* and the biosynthesis of C4-HSL also negatively affect the activity of the *pqsA* promoter	McKnight *et al*. (2000), Diggle *et al*. (2003), McGrath *et al*. (2004), Wade *et al*. (2005), Xiao *et al*. (2006a,b);
Fur and Fe^3+^	Under low iron conditions, the metabolism of anthranilate is adjusted by Fur and the PrrF sRNAs, maintaining AQ production. Iron saturation increases AQ production, probably by inducing the kynurenine pathway leading to anthranilate. Iron levels also affect the activities of AQs as inducers of PqsR	Oglesby *et al*. (2008), Hazan *et al*. (2010)
PhoB and PO_4_^3−^	AQ production is enhanced by phosphate limitation. PhoB could be mediating this by binding to a PHO box present in the *pqsR* promoter	Jensen *et al*. (2006)
PtxR	Reduces the expression of the *pqsABCDE* operon independently of *pqsR*. PtxR also acts positively on the *las* and negatively on the *rhl* QS systems	Carty *et al*. (2006)
PmpR	PmpR negatively affects the transcription of *pqsR* by binding to its promoter	Liang *et al*. (2008)
PpyR	PpyR appears to be essential for the transcription of *pqsABCDE* and *psqH*	Attila *et al*. (2008)
Dynorphin	κ-opioid receptor agonists dynorphin and U-50,488 enhance AQ production by inducing the *pqsA* promoter	Zaborina *et al*. (2007)
Farnesol	Reduces *pqsA* transcription and AQ production by interfering with PqsR	Cugini *et al*. (2007)
Indole and derivatives	Indole, its oxidation products and other bicyclic compounds, including some naphthalene analogues and 8-quinolinol, inhibit MV formation and PQS synthesis by unknown mechanisms	Tashiro *et al*. (2010)
Sputum	Growth in sputum, rich in aromatic amino acids such as tryptophan, induces the *pqsA* promoter and increases AQ production	Palmer *et al*. (2005), Farrow & Pesci (2007)

PtxR is a transcriptional regulator that positively affects the production of exotoxin A and negatively affects the production of pyocyanin. PtxR reduced the expression of the *pqsABCDE* operon, probably indirectly and not via the repression of *pqsR* (Carty *et al*., 2006). However, PtxR also regulated the *las* positively and the *rhl* QS systems negatively, which paradoxically should have resulted in an induction of the *pqsA* promoter. Therefore, it appears that PtxR could be part of an intricate network of feed-forward loops (Alon, 2007) that connect the *las*, *rhl* and *pqs* QS systems.

The gene *pmpR* (*pqsR*-mediated PQS regulator, PA0964) was found by screening transposon mutants for clones in which the *phzA1* promoter had altered expression profiles. PmpR, a protein of the YebC-like superfamily, binds to the *pqsR* promoter and affects its transcription negatively. A *pmpR* mutant was therefore found to have increased mRNA levels of *pqsR*, *pqsA* and *pqsH*, which was suggested to result in the observed induction of the *phzA1* promoter and of pyocyanin production, and enhanced swarming motility and biofilm formation (Liang *et al*., 2008).

The gene *ppyR* (*psl* and pyoverdine operon regulator, PA2663) appears to encode a membrane sensor that positively regulates exopolysaccharide and pyoverdine production, perhaps in response to the presence of NOR (Attila *et al*., 2008). The deletion of *ppyR* caused the downregulation of several genes including the *pqsABCDE* operon and the *psqH* gene for AQ and PQS biosynthesis, as well as the *antABC* operon for anthranilate degradation. As a consequence, a *ppyR* mutant produced no detectable PQS. However, the mechanism by which PpyR exerts its effects or the signals that it senses are unknown.

## Impact of AQs on microbial interactions

The prominence of *P. aeruginosa* as a major opportunistic pathogen in nosocomial infections and in lung infections in CF patients (Govan & Deretic, 1996) led to the investigation of the role of AQs in the regulation of virulence factor production and the establishment and severity of infection. The initial studies using clinical isolates from sputum in CF patients showed that they all produced PQS (Collier *et al*., 2002) and also HHQ, HQNO, NQNO and UQNO (Machan *et al*., 1992). In addition, there was a correlation between the levels of PQS and the bacterial sample load. Furthermore, PQS was also found in isolates from paediatric CF patients and from patients at early stages of *P. aeruginosa* infection (Guina *et al*., 2003). The regulation of PQS production in some of these isolates was irregular as this molecule was detected early in growth, during the log phase. The use of a simulated CF sputum medium has been shown to support the growth of *P. aeruginosa* to high population densities (Palmer *et al*., 2005) and also the differential regulation of PQS production. In particular, the expression of the *phnAB* genes is induced 14–22-fold, in line with the upregulation of the *pqsABCDE* operon (17–19-fold) compared with the expression of these genes in a morpholinepropanesulphonic acid-buffered glucose medium. This upregulation results in a fivefold increase in PQS production and presumably the other AQs, and is not triggered by changes in AHL levels. It is possibly linked to the presence of aromatic amino acids in the sputum medium such as tryptophan, which is used by *P. aeruginosa* for anthranilate biosynthesis (Farrow & Pesci, 2007). A recent study revealed that although PhhR is an aromatic amino acid-responsive transcriptional regulator that controls genes involved in phenylalanine and tyrosine catabolism in *P. aeruginosa*, the biosynthetic genes for AQs are not differentially expressed according to the presence of this regulator (Palmer *et al*., 2010).

*Pseudomonas aeruginosa* forms biofilms to protect itself from the harsh environmental conditions generated by the host immune system and antimicrobials. AQs play an important role in the establishment and maintenance of the biofilm lifestyle by a number of different mechanisms. The exogenous addition of 60 μM PQS to growing cultures of *P. aeruginosa* PAO1 resulted in a significant enhancement in biofilm formation partly due to the induction of expression of the lectin gene *lecA* (Diggle *et al*., 2003) as this gene plays a role in maintaining biofilm architecture in this organism (Diggle *et al*., 2006b). *Pseudomonas aeruginosa* can also release, possibly through lysis of cell subpopulations, extracellular DNA, which acts as an interconnecting matrix in bacterial biofilms (Whitchurch *et al*., 2002). DNA has cation-chelating and antimicrobial properties and can cause the disruption of the bacterial outer membrane by chelating Mg^2+^, which is essential for membrane stability. This in turn could result in more DNA release (Mulcahy *et al*., 2008). In addition, Mg^2+^ chelation induces the expression of the PhoPQ two-component system, increasing the resistance of *P. aeruginosa* towards aminoglycosides such as gentamicin and cationic antimicrobial peptides. These broad-spectrum antimicrobial peptides are released from host immune cells and can disrupt the bacterial outer membrane, causing cell death. Maximum DNA release takes place in the late log phase when PQS production is at its highest (Diggle *et al*., 2003; Lépine *et al*., 2003;). Similarly, a *pqsA* mutant releases low levels of extracellular DNA and forms flat, thin unstructured biofilms with increased sensitivity to detergents. The detergent sensitivity may be due to the loss of this extracellular DNA as a wild-type biofilm treated with DNase retains this sensitivity (Allesen-Holm *et al*., 2006; Häussler & Becker, 2008;). A correlation between bacterial cell lysis and PQS levels has been established, which may explain the release of the extracellular DNA observed in biofilms (D'Argenio *et al*., 2002). A mutation in the *pqsL* gene (which results in PQS overproduction) resulted in pronounced lysis in bacterial colonies, whereas those from *pqsA* and *pqsR* mutants displayed no lysis, but this could be restored upon addition of exogenous PQS. It has been proposed that PQS induces prophage-mediated lysis and that this is responsible for the DNA release (D'Argenio *et al*., 2002). The chromosome of *P. aeruginosa* harbours the filamentous Pf4 prophage, whose deletion results in the loss of bacterial autolysis and aberrant biofilm formation (Rice *et al*., 2009). PQS also acts as a pro-oxidant, which can increase the sensitivity of *P. aeruginosa* to peroxide and ciprofloxacin (Häussler & Becker, 2008), possibly resulting in cell lysis and DNA release.

AQs inhibit the growth of *S. aureus* and the yeast *C. albicans*, suggesting that they may be used as antibiotics by *P. aeruginosa*, during the early stages of infection, enabling it to eradicate any competing organisms (Machan *et al*., 1992). This idea is further supported by the fact that AQs packaged in MVs inhibited the growth of *S. epidermidis* (Mashburn & Whiteley, 2005), whereas mutants in *kynAU* were unable to kill *S. aureus* and a *kynB* mutant displayed reduced killing, presumably due to the lack of AQ production (Farrow & Pesci, 2007). As mentioned earlier (Natural antimicrobial quinolones), both HHQ and PHQ have antibacterial activities, while PHQ additionally presents antialgal properties (Wratten *et al*., 1977; Long *et al*., 2003;). HHQ and HQNO produced by a clinical isolate of *P. aeruginosa* inhibited the growth of metronidazole-resistant *H. pylori* in a cross-streak assay (Lacey *et al*., 1995). These findings may explain why early colonizers of the CF lung such as *S. aureus* are sometimes absent upon *P. aeruginosa* colonization, which outcompetes other organisms sharing the same niche (Machan *et al*., 1992). Consequently, the combined iron-chelating properties and the impact on virulence factor production of AQs help *P. aeruginosa* to generate a highly favourable environment in which to thrive.

Interestingly, farnesol, a sesquiterpene signal molecule produced by *C. albicans*, reduces the transcription of *pqsA* by interacting with PqsR and probably interfering with the normal binding of this transcriptional regulator to the *pqsA* promoter (Cugini *et al*., 2007). This results in a decrease in both PQS and pyocyanin production and suggests that this type of interspecies competition can be reciprocal. In addition, HQNO has been shown to induce the formation of persistent small-colony variants of *S. aureus* that may resist *P. aeruginosa* niche colonization and possibly explain the coexistence of these two organisms in some infections (Hoffman *et al*., 2006).

## Roles of AQs in infection

The role of AQs in virulence and the severity of infection has been demonstrated using several disease models. A mutation in *phnAB* resulted in a fourfold decrease in virulence compared with the wild type in a wax moth (*Galleria mellonella*) larvae model (Jander *et al*., 2000). In addition, mutations in *pqsC*, *pqsD*, *pqsE*, *pqsR*, *pqsH* and *phnA* resulted in severely reduced killing of the nematode (*C. elegans*) by *P. aeruginosa* to between 37 and 39% of the wild-type levels (Gallagher *et al*., 2002). Using a burned mouse model, mutants in *pqsA* and *pqsE* also exhibit reduced virulence (Déziel *et al*., 2005; Rampioni *et al*., 2010;). In the same disease model, a *pqsR* mutant showed an ∼35% reduced mortality rate compared with the wild type. This mutant also showed reduced PQS, 3-oxo-C12-HSL, pyocyanin, elastase and exoprotein production (Cao *et al*., 2001). Most interestingly, a *pqsH* mutation in *P. aeruginosa* PA14 was not attenuated, suggesting that PQS may not be essential for virulence and that virulence may be regulated via the biosynthetic precursor, HHQ (Xiao *et al*., 2006a).

Virulence factor and AQ production are upregulated in response to host stress responses to *P. aeruginosa*. The synthetic opioid U-50,488 and the endogenous κ-opioid receptor agonist dynorphin, which is released into the human small intestine during inflammation and appears to bind and enter bacterial cells, have been tested in *P. aeruginosa* PAO1 and were found to enhance virulence factor production (Zaborina *et al*., 2007). Furthermore, the addition of U-50,488 or dynorphin to a PAO1 culture induced a dose-dependent increase in pyocyanin production and enhanced *pqsA* and *lecA* (but not *pqsR*) expression, with a corresponding increase in PQS, HHQ and HQNO production. These opioid agonists also enhanced *P. aeruginosa* virulence against *Lactobacillus* and *C*. *elegans*, probably as a result of the above increases in virulence determinant production.

AQs may also interfere with host responses by acting as immune modulators. PQS suppresses T-cell proliferation and interleukin-2 release in concanavalin A-activated human peripheral blood mononuclear cells (hPBMCs). PQS also induces tumour necrosis factor-α release from lipopolysaccharide-activated hPBMCs, at concentrations around 10 μM (Hooi *et al*., 2004). *In vitro*, PQS reduces the release of interleukin-12 from lipopolysaccharide-stimulated bone marrow-derived dendritic cells, preventing the development of naïve T cells into T-helper type 1 cells, which promote cell-mediated immunity. The concentration of PQS required to lower the cytokine release to 50% in this case was below 20 μM (Skindersoe *et al*., 2009). Additionally, both HHQ and PQS appear to suppress host innate immune systems by interfering with the nuclear transcription factor-κB signalling pathway. This effect can be achieved with cell-free extracts from cultures of wild-type *P. aeruginosa*, but not from the cultures of a corresponding *pqsA* mutant (Kim *et al*., 2010). It therefore seems possible that AQs play a role in the dysregulation of the host immune response during infection.

## Production of AQs by other bacteria

DNA database analysis has revealed the presence of *pqs* gene homologues in >40 species and strains that are more or less related to *P. aeruginosa*. In particular, *Burkholderia pseudomallei* and *B. thailandensis* appear to have the complete putative *pqsABCDE* operons in their chromosomes, sharing 31–53% identity to that of *P. aeruginosa* (Diggle *et al*., 2006a). These were named *hhqABCDE* as no PQS had been detected in these organisms. The *hhqA* and *hhqE* genes are functionally conserved with their *P. aeruginosa* homologues as they were able to complement PAO1 *pqsA* and *pqsE* mutants, respectively, and restore PQS, HHQ, lectin and pyocyanin production in the *pqsA* mutant and pyocyanin and lectin production in the *pqsE* mutant. Using a combination of a novel AQ bioreporter and LCMS/MS, HHQ was detected in culture supernatants of *Pseudomonas putida* and *Burkholderia cenocepacia* and HHQ, NHQ, UHQ and HQNO in *B. pseudomallei* (Diggle *et al*., 2003). Although a mutant unable to generate AQs in *B. pseudomallei* presented altered colony morphology and increased elastase production, the actual role of these molecules in the biology of this organism remains to be unravelled. AQs have also been identified in a number of species of *Burkholderia* such as *Burkholderia ambifaria*, *B. thailandensis*, *B. pseudomallei* and *Pseudomonas cepacia* (probably an unclassified *Burkholderia*). The main AQs produced by these organisms are 3-methyl derivatives of PHQ, HHQ and NHQ termed 4-hydroxy-3-methyl-2-alkylquinolines (Moon *et al*., 1996; Vial *et al*., 2008;). Consequently, the operon responsible for their synthesis has been renamed *hmqABCDEFG* (formerly *hhqABCDE*), with the predicted methyltransferase *hmqG* being involved in the biosynthesis of these AQs. None of the above bacteria has *pqsH* orthologues nor produces PQS, and previous efforts to detect PQS in other pseudomonads such as *P. fluorescens*, *Pseudomonas syringae* and *Pseudomonas fragi* have been unsuccessful (Lépine *et al*., 2004). Furthermore, in *B. thailandensis*, *B. ambifaria* and *B. pseudomallei*, the −3′ position is largely methylated (Vial *et al*., 2008), which would presumably preclude any *pqsH* analogue hydroxylating in these molecules and hence the production of PQS. These findings suggest that these organisms may lack the complexity of PQS signalling found in *P. aeruginosa*.

## Concluding remarks

The discovery of quinine and related natural antiplasmodial alkaloids, combined with the advances of synthetic chemistry, spurred significant research in the field and resulted in the development and evaluation of thousands of novel synthetic compounds. Among these were nalidixic acid and the extensive family of synthetic quinolone antibiotics. In parallel, the quest for antimicrobials of natural origin lead to the discovery of the AQNOs and of the extensive family of AQ compounds mostly produced by *P. aeruginosa* and related bacteria. Synthetic and natural quinolone antimicrobials, however, appear to share little in common with respect to their mode of action. The biological roles of the natural quinolones of bacterial origin are diverse and include intercellular signalling. The discovery of a non-AHL-based QS system in *P. aeruginosa* mediated via AQs and linked to the *las* and *rhl* QS systems provides a major insight into a complex regulatory network that plays key roles in infection via the regulation of virulence and biofilm maturation. Some AQs, such as PQS, are also able to sequester iron and have multiple functionalities. AQ biosynthesis requires several proteins and occurs via a condensation reaction between anthranilate and β-keto fatty acids. Their production is upregulated by both the *las* QS system and by AQs themselves and downregulated by the *rhl* QS system. AQs are present in bacteria other than *P. aeruginosa*, mainly other pseudomonads and *Burkholderiaceae*, although the role in these organisms is not, at present, very well defined. It is possible that AQs could be produced by many other bacterial genera, as the number of studies where these compounds have been specifically screened has been small and yet several AQs-producing species have been discovered (Lépine *et al*., 2004; Diggle *et al*., 2006a; Vial *et al*., 2008;). In most of the species that produce AQs, the role of these compounds or their biosynthesis remains unclear, although for many where genomic sequences are available, orthologues of the *pqsABCDE* operon extensively studied in *P. aeruginosa* can often be identified.

Quorum quenching is the process by which the signalling mediating QS is interfered with, leading to the disruption of the normal means by which bacteria coordinate their behaviour according to their population density and preventing colonization (Raina *et al*., 2009). Quorum quenching can be exerted naturally by microorganisms to prevent the establishment of competing species and offers a strategy for the development of novel antimicrobial drugs (Rasmussen & Givskov, 2006). Hence, quinolone quenching offers the possibility to interfere with AQ signalling in pathogens such as *P. aeruginosa*, which rely on it to control virulence. As a primary target to interfere with AQ-mediated signalling, binding of PQS and HHQ to PqsR could be blocked. This would not only interfere with the production of AQs and their associated properties beneficial for the bacterial cell by preventing the expression of the *pqsABCDE* operon, but would also downregulate all the other AQ-regulated genes, including those essential for virulence. Compounds such as farnesol inhibit the induction capacity of PqsR on the *pqsA* promoter (Cugini *et al*., 2007). However, as these compounds were found to act only at relatively high concentrations (in the mM range), other, more potent inhibitors are needed to stimulate clinical interest. Another possibility would be to inhibit the action of PqsE, whose function is still unclear, but that is required for the production of several virulence factors. Analogues of AQ precursors such as methyl-anthranilate or halogenated derivatives of anthranilate have been found to inhibit AQ synthesis, thus interfering with the signalling system probably by acting as competitive inhibitors of PqsA (Calfee *et al*., 2001; Lesic *et al*., 2007; Coleman *et al*., 2008;). This approach has recently shown promising results in limiting the systemic proliferation of *P. aeruginosa* infection in mice (Lesic *et al*., 2007).

To complete our understanding of the AQ signalling system in *P. aeruginosa*, the roles of some components still remain to be fully unravelled. PqsB, PqsC and PqsL are likely to be involved in the biosynthesis of AQs as revealed by the structural domains they share with other known enzymes and by mutational analysis (Gallagher *et al*., 2002; Diggle *et al*., 2003; Bredenbruch *et al*., 2005; Farrow *et al*., 2008;). However, the exact role of PqsB and PqsC in the biosynthesis of HHQ and PQS still remains unclear. Similarly, little is known about the interaction of PqsL with precursor and other biosynthetic proteins to generate the *N*-oxide AQNOs, because the otherwise common precursor molecule HHQ does not appear to be required for their production (Déziel *et al*., 2004). Even more critically, the role played by PqsE, which appears to be an enzyme of the metallo-β-hydrolase superfamily mediating the signal transduction that upregulates swarming motility, the production of pyocyanin, lectin, HCN, the transcription of many genes and ultimately virulence (Rampioni *et al*., 2010) remain to be elucidated.

The biology of AQ biosynthesis, its regulation and the signalling functions of the AQs control the behaviour and virulence of *P. aeruginosa*. AQ signalling is proving to be complex, leading to many open questions that still remain unanswered. For example, the biological roles of AQs such as DHQ or the *N*-oxide derivatives in *P. aeruginosa* remain to be elucidated, as are the functions of AQs in other pathogenic and beneficial microorganisms producing them. From their inconspicuous discovery as potentially useful compounds having weak antimicrobial properties, quinolones in general and AQs in particular are highly versatile molecules that play central roles in the biology of the producer organisms.

## Statement

Re-use of this article is permitted in accordance with the Terms and Conditions set out at http://wileyonlinelibrary.com/onlineopen#OnlineOpen_Terms
